# Social Media Addiction and Loneliness Among Individuals Undergoing Substance Use Rehabilitation: The Role of Demographics

**DOI:** 10.3390/healthcare14203540

**Published:** 2026-10-21

**Authors:** Azyyati Mohd Suhaimi, Kang Wan Ru, Umar Idris Ibrahim, Nor Hayati Abu Samah, Shazia Jamshed, Ayesha Iqbal, Mohamed N. Al Arifi, Mohammad K. Alharbi, Naji Alqahtani, Adel Bashatah, Saeed Asiri, Wajid Syed

**Affiliations:** 1 Department of Basic Medical Science and Pharmacology, School of Pharmacy, Management and Science University, University Drive, Off Persiaran Olahraga, Section 13, Shah Alam 40100, Selangor, Malaysia; 2 Department of Clinical Pharmacy and Pharmacy Practice, Faculty of Pharmacy, Universiti Sultan Zainal Abidin, Kampus Besut, Besut 22200, Terengganu, Malaysia; 3 Department of Pharmaceutics, Faculty of Pharmacy, Universiti Teknologi MARA, Puncak Alam Campus, Puncak Alam 42300, Selangor, Malaysia; norhay2975@uitm.edu.my; 4 Department of Pharmacy Practice, School of Pharmacy, IMU (Former International Medical University), Kuala Lumpur 57000, Malaysia; shaziajamshed@imu.edu.my; 5 Department of Pharmacy Practice and Policy, University Park Campus, University of Nottingham, Nottingham NG72RD, UK; 6 Office of Lifelong Learning and the Physician Learning Program, Faculty of Medicine and Dentistry, University of Alberta, Edmonton, AB T6G1C9, Canada; 7 Department of Clinical Pharmacy, College of Pharmacy, King Saud University, Riyadh 11451, Saudi Arabia; 8 Department of Nursing Administration and Education, College of Nursing, King Saud University, Riyadh 11451, Saudi Arabia

**Keywords:** social media addiction, loneliness, substance use

## Abstract

**Background**: Social media addiction and loneliness are important psychosocial concerns among individuals undergoing rehabilitation for substance use disorders. This study examined the levels of social media addiction and loneliness and their relationship among residents of Malaysian rehabilitation centers. **Methods**: A cross-sectional study was conducted among individuals undergoing rehabilitation at three PUSPEN centers in Kelantan and Terengganu, Malaysia. The Bergen Social Media Addiction Scale (BSMAS) and 8-item UCLA Loneliness Scale (UCLA-8) were administered. Associations were examined using Spearman’s correlation and multiple linear regression. **Results**: The mean age of participants was 26.53 ± 6.59 years. The mean BSMAS score was 16.12 ± 4.94, with mild social media addiction being the most common category (41.9%), followed by low (27.1%), moderate (18.6%), and high (12.4%) levels. The mean UCLA-8 loneliness score was 16.91 ± 5.63, with 44.2% reporting moderate loneliness, 41.9% low loneliness, and 14.0% high loneliness. Continuous BSMAS scores were weakly and positively correlated with UCLA-8 scores (r_s_ = 0.211, *p* = 0.016). In the adjusted regression model, female gender was significantly associated with higher loneliness scores (B = 3.861, *p* = 0.015), whereas social media addiction was no longer significant (B = 0.151, *p* = 0.134). **Conclusions**: While mild social media addiction and moderate loneliness are common among individuals in substance rehabilitation, feeling lonely is more closely linked to baseline demographic characteristics than to pre-admission digital habits. Because accounting for gender substantially reduces the apparent connection between digital addiction and isolation, institutional policies should move beyond simple device restrictions to address residents’ underlying psychosocial needs.

## 1. Introduction

Substance use disorder (SUD) remains a major public health concern, particularly among adolescents and young adults. Individuals with SUD often experience adverse physical, psychological, and social consequences that can impede recovery and reintegration into society. In Malaysia, young adults aged 19–39 years constitute the largest proportion of individuals identified with drug use problems, with methamphetamine and cannabis among the most commonly abused substances [1,2]. To address substance use disorders, Malaysia has established treatment and rehabilitation services administered by the National Anti-Drugs Agency (NADA). The agency operates under the Ministry of Home Affairs and is responsible for drug prevention, treatment, and rehabilitation initiatives. Pusat Pemulihan Penagihan Narkotik (PUSPEN) centers provide institutional treatment and rehabilitation services within this framework, with voluntary treatment services also available under the Drug Dependants (Treatment and Rehabilitation) Act 1983 (Act 283) [3,4].

Alongside substance use, problematic engagement with digital technologies has emerged as a growing psychosocial concern. Malaysia is among the countries with the highest levels of internet and social media engagement, with social networking platforms such as TikTok, Instagram, Facebook, and X forming an integral part of daily life [5]. While social media offers opportunities for communication, information sharing, and social support, excessive and uncontrolled use may develop into social media addiction, a behavioral condition characterized by salience, mood modification, tolerance, withdrawal, conflict, and relapse [6,7]. Increasing evidence suggests that problematic social media use is associated with a range of negative psychological outcomes, including anxiety, depression, poor well-being, and impaired social functioning.

Loneliness is another important psychosocial issue affecting individuals undergoing rehabilitation for substance use disorders. Defined as the subjective discrepancy between desired and actual social relationships, loneliness has been consistently linked to poorer mental health outcomes and reduced quality of life [8,9]. Individuals with substance use disorders are particularly vulnerable to loneliness due to social stigma, fractured family relationships, disrupted peer networks, and prolonged periods of institutionalization. At the same time, loneliness may function as a risk factor for maladaptive coping behaviours, including excessive social media use and substance use. Previous studies have reported significant associations between problematic social media use and loneliness among adolescents and young adults, suggesting that individuals may turn to online platforms to compensate for unmet social and emotional needs [8,10,11].

The relationship between loneliness, social media addiction, and substance use may be explained by the Compensatory Internet Use Theory, which proposes that individuals engage excessively in online activities to alleviate negative emotions and psychosocial distress [12]. For individuals undergoing substance abuse rehabilitation, social media may serve as an accessible means of seeking connection and emotional support. However, excessive reliance on social media may paradoxically reinforce feelings of isolation and contribute to the development of problematic online behaviours. Furthermore, people with substance use disorders have been reported to exhibit higher levels of loneliness and greater susceptibility to behavioral addictions compared with the general population [13,14].

Despite increasing interest in the psychological effects of social media use, empirical evidence examining the relationship between social media addiction and loneliness among individuals with substance use disorders in Malaysia remains limited. Understanding this relationship is particularly important within rehabilitation settings, where psychosocial well-being plays a crucial role in treatment engagement and long-term recovery outcomes. Therefore, this study aimed to determine the levels of social media addiction and loneliness and to examine the relationship between social media addiction and loneliness among residents undergoing substance abuse rehabilitation at selected PUSPEN centers in Malaysia.

## 2. Methods

### 2.1. Study Design and Sample Recruitment

A cross-sectional descriptive–correlational study was conducted between April and June 2026 among clients undergoing rehabilitation at three NADA rehabilitation centers, namely PUSPEN Besut, PUSPEN Bachok, and PUSPEN Kota Bharu, Malaysia, through statutory admission pathways under the Drug Dependants (Treatment and Rehabilitation) Act 1983. Female clients were enrolled at PUSPEN Bachok, while male clients were recruited across PUSPEN Besut and Kota Bharu due to gender-segregated residential policies in Malaysia. Participants were eligible for inclusion if they were adults aged 18–49 years, were able to understand and complete the study questionnaire, and could communicate in the Malay language, either verbally or in writing. To ensure participants had sufficiently adapted to the rehabilitation environment and were emotionally stable, only individuals who had been enrolled in the rehabilitation programme for more than two weeks were recruited. Participants with severe psychological or cognitive impairments that could affect their ability to comprehend or respond to the questionnaire, including intellectual disability, dementia, or acute schizophrenia, were excluded. Individuals who declined participation, did not provide informed consent, had been in rehabilitation for less than two weeks, or submitted incomplete questionnaires were also excluded from the study. Participants were recruited using a convenience sampling approach with the assistance of PUSPEN officers during scheduled rehabilitation activities. A total of 140 eligible residents were approached, of whom 130 completed the questionnaire (92.9% response rate). One respondent was excluded due to an unresolvable item–total sum discrepancy, resulting in a final analyzed sample of N = 129.

### 2.2. Sample Size

Sample size requirements were determined a priori using G*Power software (version 3.1.9.7). For a multivariable linear regression model with three predictors (age, social media addiction, and gender), a minimum sample size of N = 77 was required to detect a standard medium effect size (f2 = 0.15) at α = 0.05 with 80% statistical power (1 − β = 0.80). Out of 140 clients approached across three rehabilitation facilities, 129 were included in the final analysis (response rate = 92.1%). A post-hoc power analysis indicated that the final sample of N = 129 achieved a statistical power of 76.7% for the multivariable model (R2 = 0.075, f2 = 0.0811, α = 0.05).

### 2.3. Data Collection and Study Instruments

Data collection was conducted at the participating rehabilitation centers through either small-group or individual sessions, depending on participant availability and programme schedules. Prior to participation, all eligible individuals received a standardized explanation of the study objectives, procedures, confidentiality measures, and their right to withdraw without penalty. Written informed consent was obtained from all participants before data collection commenced. The self-administered questionnaire, available in the Malay language, consisted of three sections: sociodemographic characteristics, social media addiction, and loneliness. Social media addiction was measured using the 6-item Bergen Social Media Addiction Scale [15]. Each item is rated on a 5-point Likert scale (1 = Very rarely to 5 = Very often), yielding a total score range of 6 to 30. The Malay version of the BSMAS was validated by Tiong (2020) [16,17], demonstrating good internal consistency (Cronbach’s α = 0.800), unidimensional factor structure via Confirmatory Factor Analysis (CFI = 1.000, TLI = 1.000, RMSEA < 0.001), and high concurrent validity (r = 0.559). The total BSMAS scores were categorized into four severity intervals: Low (6–11), Mild (12–17), Moderate (18–23), and High (24–30) addiction severity [16,18]. Perceived loneliness was evaluated using the 8-item University of California, Los Angeles Loneliness Scale [19]. Items are rated on a 4-point Likert scale (1 = Never to 4 = Always), producing a total score range of 8 to 32 after reverse-scoring items 3 and 6. The Malay version of the UCLA-8 was validated by Swami (2009), exhibiting excellent internal reliability (Cronbach’s α = 0.85) among Malaysian cohorts [20]. For descriptive purposes, UCLA-8 scores were grouped into three loneliness tiers: Low (8–15), Moderate (16–23), and High (24–32) loneliness [19,20]. For the present study, the calculated Cronbach’s α values for BSMAS and UCLA-8 were 0.769 and 0.679 respectively.

### 2.4. Statistical Analysis

Data were analyzed using IBM SPSS Statistics version 30.0. Prior to analysis, data were screened for completeness and accuracy. Scale scores were verified by recalculating total scores from individual item responses according to the respective scoring guidelines, including reverse scoring of the negatively worded items in the UCLA-8 Loneliness Scale. Descriptive statistics were used to summarize participant characteristics and study variables. Categorical variables were presented as frequencies and percentages, whereas continuous variables were summarized using means, standard deviations (SD), medians, ranges, and skewness values. Based on established scoring criteria, BSMAS scores were categorized into low, mild, moderate, and high levels of social media addiction, while UCLA-8 scores were categorized into low, moderate, and high levels of loneliness. Relationships among age, social media addiction, and loneliness were examined using Spearman’s rank-order correlation coefficient (rs).

To identify correlates of loneliness, multiple linear regression based on the ordinary least squares (OLS) method was conducted, with UCLA-8 total score as the dependent variable. In Model 1, age and social media addiction (BSMAS total score) were entered simultaneously as independent variables. In Model 2, gender was added as a control variable to assess its contribution beyond age and social media addiction. Gender was dummy coded, with males coded as 0 and females coded as 1. For each model, unstandardized regression coefficients (B), standard errors (SE), t-values, *p*-values, coefficients of determination (R^2^ and adjusted R^2^), and overall model F-statistics were reported. Participant age was incorporated as a primary demographic covariate in multivariable linear regression modeling (Model 2) to control for baseline demographic imbalances across gender groups. Collinearity diagnostics, including Variance Inflation Factors (VIFs), were examined to assess model stability. A two-tailed *p*-value of < 0.05 was considered statistically significant for all analyses

### 2.5. Ethical Considerations

Ethical approval for the study was obtained from the Universiti Sultan Zainal Abidin Human Research Ethics Committee (UniSZA/UHREC/2026/990) dated on 19 February 2026, prior to data collection. Participation was voluntary, and all data were collected anonymously and handled confidentially throughout the study. Informed consent was obtained prior to data collection and participants were informed about the confidentiality and data would be used only for the research and they have the rights withdraw from the study at any point of time and all the study procedures were performed according to Declaration of Helsinki guidelines for human research.

## 3. Results

### 3.1. Sociodemographic and Sample Characteristics

The sample was predominantly male (65.1%, *n* = 84), with female clients constituting 34.9% (n = 45) of the cohort. The overall mean age of the respondents was 26.53 ± 6.59 years. However, male clients had a mean age of 22.94 ± 2.27 years, whereas female clients had a mean age of 33.27 ± 6.72 years, indicating a baseline age disparity (Table 1).

### 3.2. Prevalence and Severity of Social Media Addiction and Loneliness

The sample-wide mean score for social media addiction was 16.12 ± 4.94 (Table 2). Categorical severity distributions based on BSMAS diagnostic guidelines revealed that 41.9% (n = 54) of respondents experienced mild social media addiction, followed by low addiction (27.1%, n = 35), moderate addiction (18.6%, n = 24), and high addiction levels (12.4%, n = 16). For perceived loneliness, the overall mean score was 16.91 ± 5.63. Most of them fell into the moderate loneliness category (44.2%, n = 57), followed closely by low loneliness (41.9%, n = 54) and high loneliness (14.0%, n = 18). Taken together, these descriptive patterns indicate that mild social media addiction and moderate loneliness constitute the primary psychosocial profiles within this rehabilitation cohort.

### 3.3. Bivariate Correlations

At the continuous level, a bivariate Spearman’s rank-order correlation analysis (Table 3) demonstrated a statistically significant, weakly positive correlation between continuous BSMAS and UCLA-8 total scores (rs = 0.211, *p* = 0.016). No statistically significant correlations were found between participant age and social media addiction (rs = −0.053, *p* = 0.553) or perceived loneliness (rs = −0.066, *p* = 0.456). [Note: The categorical Chi-Square analysis has been removed to rely exclusively on continuous scale scores.]

### 3.4. Multiple Linear Regression

In the initial regression model (Model 1), social media addiction demonstrated a weak positive association with loneliness that approached statistical significance (B = 0.200, SE = 0.100, *p* = 0.048, β = 0.175). Following the addition of gender in Model 2, the unstandardized coefficient for social media addiction was attenuated to B = 0.151 (*p* = 0.134), while female gender emerged as an independent demographic correlate of institutional loneliness (B = 3.861, SE = 1.558, *p* = 0.015, β = 0.328) (Table 4). This represents a 21.3% reduction in magnitude for the BSMAS coefficient. Such partial attenuation demonstrates that while social media addiction shares a modest bivariate overlap with loneliness, female gender represents an independent demographic correlate of institutional loneliness. Controlling for age was essential due to a substantial baseline age disparity within this residential rehabilitation cohort. Because age demonstrated an inverse partial relationship with loneliness (B = −0.198, SE = 0.114, *p* = 0.083), older age exerted a protective effect that masked baseline loneliness scores among female residents in raw bivariate comparisons. Adjusting for age held participant age constant across gender groups, thus removing age-related variance and isolating the unconfounded independent association between female gender and institutional loneliness. Collinearity diagnostics confirmed model stability, with Variance Inflation Factors for all predictors ranging between 1.01 and 1.02.

## 4. Discussion

The current study represents an assessment of the co-occurring challenges of social media addiction and perceived loneliness among individuals undergoing institutional substance use rehabilitation in Malaysia. While our findings support the consensus that mild-to-moderate levels of both social media addiction and loneliness are prevalent in this population, our advanced multivariable modeling reveals a much more nuanced psychosocial structure than previously reported in bivariate studies. When controlling for participant gender, the independent association of social media addiction with loneliness is attenuated, while female gender emerges as a primary demographic correlate of subjective isolation. In this sample, gender is associated with loneliness after adjustment for the variables included in the model, suggesting that the apparent behavioral overlap between social media use and loneliness is heavily influenced by demographic baseline isolation.

Under the Compensatory Internet Use Theory, lonely individuals are thought to turn to digital spaces to alleviate negative affect and satisfy unmet interpersonal needs [12,21,22]. However, our multiple regression analysis challenges this direct path. When controlling for participant gender, the independent effect of social media addiction on loneliness completely disappears (*p* = 0.134), while female gender emerges as the primary, highly significant predictor of subjective isolation (*p* = 0.012). This suggests that in a structured rehabilitation setting, the correlation between social media use and loneliness is heavily confounded. Female individuals report both higher subjective loneliness and slightly elevated digital addiction scores. When these variables are analyzed simultaneously, the apparent behavioral effect of social media is shown to be a byproduct of gender-specific baseline isolation. This finding is compatible with the possibility that social media use may function as a compensatory response to psychosocial distress; however, the cross-sectional design does not allow the direction of this relationship to be established.

The significantly higher loneliness scores reported by female clients point to a severe psychosocial gap in the Malaysian rehabilitation framework. This acute isolation is highly consistent with the work of Greenfield et al. who noted that women entering substance treatment carry disproportionate burdens of societal stigma, acute family disruption, fractured maternal ties, and histories of physical or emotional trauma [23,24,25]. In this country, substance use has historically been addressed through punitive and criminal justice approaches, contributing to substantial stigma toward people who use drugs. Women who use drugs often experience a particularly heightened form of social marginalization because their substance use is perceived not only as a violation of legal and social norms but also as a transgression of culturally prescribed gender, familial, and caregiving roles. As a result, women may face greater social disapproval, discrimination, and exclusion than their male counterparts. This gendered stigma can have important psychosocial consequences [26,27]. Qualitative research among Malaysian women who use drugs has highlighted experiences of familial instability, disrupted social relationships, loss of parental roles, and limited access to supportive networks. Such experiences may contribute to greater social isolation and feelings of loneliness, particularly during treatment and rehabilitation, when individuals are separated from their usual social environments.

In contrast to studies in the general population where younger cohorts display significantly higher rates of both digital addiction and loneliness, participant age in this study was entirely independent of both constructs [28,29,30]. This lack of association is highly likely to be a reflection of the institutional setting itself. Within PUSPEN facilities managed by NADA, regardless of age, all residents are subject to identical daily routines, highly structured treatment programs, and strict limitations on physical and digital contact with the outside world. This uniform environmental context effectively normalizes psychosocial experiences, flattening the age-based variations typically observed in the community.

### Methodological Limitations and Clinical Implications

Several study limitations warrant consideration. First, the cross-sectional design impedes causal inferences regarding digital habits and loneliness. Second, convenience sampling across PUSPEN centers limits generalizability to private or community recovery settings. Third, while the overall sample (N = 129) achieved adequate statistical power, the smaller female cohort (n = 45) reflects national institutional demographics but requires cautious subgroup interpretation. Fourth, reliance on self-reported psychometric scales (BSMAS and UCLA-8) introduces potential recall and social desirability biases within a structured residential environment. A notable limitation is the absence of detailed clinical variables, such as the primary substance of abuse, duration and severity of substance use, number of previous rehabilitation admissions, exact length of current stay, and co-occurring non-acute psychiatric conditions, as well as the use of psychotropic medications. Although individuals with acute, severe cognitive or psychotic impairments were excluded from participation, unmeasured psychiatric co-morbidities and addiction characteristics could introduce residual confounding, as these clinical factors plausibly correlate with both subjective loneliness and digital media engagement. Participant age was incorporated as a primary demographic covariate in multivariable linear regression modeling (Model 2) to control for baseline demographic imbalances across gender groups.

These findings offer insights for institutional rehabilitation practices and future research. Blanket digital device restrictions in rehabilitation centers are unlikely to reduce loneliness, as isolation is primarily shaped by gender-specific vulnerabilities rather than pre-admission digital media engagement. Instead, clinical frameworks for female residents should prioritize trauma-informed care, family connection, and social network restoration. Future research should engage prospective longitudinal and mixed-methods designs incorporating key clinical variables and medication profiles to gender-tailored psychosocial interventions across treatment stages.

## 5. Conclusions

While mild social media addiction and moderate loneliness are common among individuals in substance rehabilitation, feeling lonely is more closely linked to baseline demographic characteristics than to pre-admission digital habits. Because accounting for gender substantially reduces the apparent connection between digital addiction and isolation, institutional policies should move beyond simple device restrictions to address residents’ underlying psychosocial needs. Rehabilitation program enhancements should focus on gender-sensitive and trauma-informed counselling, peer-led support groups, and family reunification strategies designed to rebuild supportive social networks.

## Figures and Tables

**Table 1:** Demographic profiles and gender-based differences.

Characteristic/Variable	Total (n = 129)	Male (n = 84)	Female (n = 45)
Age (years), mean ± SD	26.53 ± 6.59	22.94 ± 2.27	33.27 ± 6.72
Gender, n (%)		84 (65.1%)	45 (34.9%)
BSMAS total score, mean ± SD	16.12 ± 4.94	15.92 ± 4.75	16.49 ± 5.32
UCLA-8 total score, mean ± SD	16.91 ± 5.63	16.23 ± 5.58	18.20 ± 5.54

**Table 2:** BSMAS and UCLA-8 mean scores and severity levels.

Variable/Level	Frequency (n)	Percentage (%)	Mean ± SD
Social media addiction (BSMAS)			16.12 ± 4.94
• Low SMA	35	27.1%	
• Mild SMA	54	41.9%	
• Moderate SMA	24	18.6%	
• High SMA	16	12.4%	
Loneliness (UCLA-8)			16.91 ± 5.63
• Low loneliness	54	41.9%	
• Moderate loneliness	57	44.2%	
• High loneliness	18	14.0%	

**Table 3:** Spearman’s rank-order correlation matrix among participant age, social media addiction and loneliness.

Measure/Variable	Age	BSMAS Total Score	UCLA-8 Loneliness Score
Age (Correlation)	1	−0.053	−0.066
Sig. (2-tailed)		0.553	0.456
BSMAS total (Correlation)	−0.053	1	0.211 *
Sig. (2-tailed)	0.553		0.016
UCLA-8 (Correlation)	−0.066	0.211 *	1
Sig. (2-tailed)	0.456	0.016	

Note: *p* < 0.05 (two-tailed). BSMAS = Bergen Social Media Addiction Scale; UCLA-8 = 8-item UCLA Loneliness Scale; * = Correlation between the BSMAS total score (social media addiction) and the UCLA-8 loneliness score is statistically significant because *p* = 0.016.

**Table 4:** Sequential ordinary least squares (OLS) linear regression models predicting subjective loneliness.

Model/Predictor	B	SE	Beta (β)	*p*-Value	95% CI	VIF
Model 1: Age	0.019	0.075	0.022	0.799	[−0.130, 0.168]	1.00
Model 1: BSMAS total	0.200	0.100	0.175	0.048	[0.001, 0.398]	1.00
Model 2: Age	−0.198	0.114	−0.231	0.083	[−0.423, 0.027]	1.02
Model 2: BSMAS total	0.151	0.100	0.132	0.134	[−0.047, 0.348]	1.01
Model 2: Female gender	3.861	1.558	0.328	0.015	[0.778, 6.945]	1.02

## Data Availability

The datasets used or analysed during the study are available from the corresponding author upon acceptable request.
